# Production of Sucrolytic Enzyme by *Bacillus licheniformis* by the Bioconversion of Pomelo Albedo as a Carbon Source

**DOI:** 10.3390/polym13121959

**Published:** 2021-06-13

**Authors:** Chien Thang Doan, Thi Ngoc Tran, Thi Thanh Nguyen, Thi Phuong Hanh Tran, Van Bon Nguyen, Trung Dung Tran, Anh Dzung Nguyen, San-Lang Wang

**Affiliations:** 1 Department of Chemistry, Tamkang University, New Taipei City 25137, Taiwan; dcthang@ttn.edu.vn (C.T.D.); ttngoc@ttn.edu.vn (T.N.T.); 2 Faculty of Natural Sciences and Technology, Tay Nguyen University, Buon Ma Thuot 630000, Vietnam; ntthanh@ttn.edu.vn (T.T.N.); ttphanh@ttn.edu.vn (T.P.H.T.); ttdung@ttn.edu.vn (T.D.T.); 3 Institute of Biotechnology and Environment, Tay Nguyen University, Buon Ma Thuot 630000, Vietnam; nvbon@ttn.edu.vn (V.B.N.); nadzung@ttn.edu.vn (A.D.N.); 4 Life Science Development Center, Tamkang University, New Taipei City 25137, Taiwan

**Keywords:** agro-byproduct, *Bacillus licheniformis*, bioconversion, pomelo albedo, sucrolytic

## Abstract

Recently, there has been increasing use of agro-byproducts in microbial fermentation to produce a variety of value-added products. In this study, among various kinds of agro-byproducts, pomelo albedo powder (PAP) was found to be the most effective carbon source for the production of sucrose hydrolyzing enzyme by *Bacillus licheniformis* TKU004. The optimal medium for sucrolytic enzyme production contained 2% PAP, 0.75% NH_4_NO_3_, 0.05% MgSO_4_, and 0.05% NaH_2_PO_4_ and the optimal culture conditions were pH 6.7, 35 °C, 150 rpm, and 24 h. Accordingly, the highest sucrolytic activity was 1.87 U/mL, 4.79-fold higher than that from standard conditions using sucrose as the carbon source. The purified sucrolytic enzyme (sleTKU004) is a 53 kDa monomeric protein and belongs to the glycoside hydrolase family 68. The optimum temperature and pH of sleTKU004 were 50 °C, and pH = 6, respectively. SleTKU004 could hydrolyze sucrose, raffinose, and stachyose by attacking the glycoside linkage between glucose and fructose molecules of the sucrose unit. The *K_m_* and *V_max_* of sleTKU004 were 1.16 M and 5.99 µmol/min, respectively. Finally, sleTKU004 showed strong sucrose tolerance and presented the highest hydrolytic activity at the sucrose concentration of 1.2 M–1.5 M.

## 1. Introduction

Sucrose (*α*-D-glucopyranosyl-1,2-*β*-D-fructofuranoside), the world’s most abundant disaccharide, comprises a glucose unit and a fructose unit linked together by an *α*1–*β*2 linkage [1]. Sucrose hydrolysis is catalyzed by sucrolytic enzymes to produce a mixture of glucose and fructose (also known as invert sugar). Sucrolytic enzymes are of various types such as invertases (sucrases) [2], levansucrases [3], dextransucrases [4], glucansucrases [5], and inulosucrases [6]. They use sucrose as the donor of fructosyl moiety to be transferred to various kinds of acceptors, such as water (sucrose hydrolysis), sucrose, and fructan [7]. Therefore, these enzymes are also responsible for the production of some compounds such as fructooligosaccharides (FOSs), inulins, and levans [8,9]. To date, sucrolytic enzymes have diverse applications in the food [10,11,12], beverage [13,14], medicine [1,14], cosmetic [5,15], bioenergy [16], paper [15], and biochemical sectors [15]. Thus, the use of sucrolytic enzymes is highly widespread, with a large number of industrial applications.

The worldwide market for enzymes is anticipated to reach $10.5 billion in 2024 [17]. Among these, enzymes from microbial sources are the most important group, comprising 90% of the global market [18]. Regardless of their high demand, the price of enzymes still remains relatively high, which thus increases the cost of the processes using enzymatic methods. Therefore, for the cost-effective production of enzymes, several strategies are being developed including using cheap and readily available carbon and nitrogen sources such as the wastes from food processing, fishery, and agriculture as the alternative substrates for the microbial fermentation process [19,20,21,22]. Besides, the utilization of by-products for enzyme production is also an environmentally friendly technique because it can reduce the amount of waste generated and released into the environment [23]. Accordingly, this green technique has received a great attention, and as a result, various kinds of enzymes have been effectively produced by using by-products as the major nutrient source such as proteases [22,24], xylanases [21,25], chitosanases [26,27], chitinases [28,29], pectinases [23,30], invertases [31,32], etc.

*Bacillus licheniformis* is a rod-shaped, facultatively anaerobic, Gram-positive, and spore-forming bacterium [33]. *B. licheniformis* is favored because of its outstanding fermentation properties, high protein yield, and completely toxin-free productivity [34]. To date, various applications of *B. licheniformis* have been explored for the production of probiotics [35], industrial enzymes [36,37], and biofuels [37]. *B. licheniformis* has been commonly used for the manufacture of amylase and protease [37,38,39,40,41], however, the production of sucrolytic enzymes by this genus has not been fully explored. In fact, there are only a few reports on the production of sucrose hydrolyzing enzymes by *B. licheniformis*, especially using low-cost materials for the fermentation process [42,43,44]. In comparison, several *B. licheniformis* strains are known to have a great capacity for processing agricultural byproducts to produce enzymes [39,45]. This leads to the idea of using agro-byproducts as economically effective nutritional supplements for the synthesis of sucrose hydrolytic enzymes by *B. licheniformis*.

The exploration of new enzymes, having a low-cost manufacturing method, and possessing desirable characteristics, has always been regarded as important. Accordingly, in this study, various kinds of agro-byproducts such as pomelo (*Citrus maxima*) albedo powder (PAP), pomelo flavedo powder (PFP), orange (*Citrus sinensis*) peel powder (OPP), banana (*Musa paradisiaca*) peel powder (BPP), rice bran powder (RBP), and wheat bran powder (WBP) were used as carbon sources for *B. licheniformis* TKU004 to produce sucrolytic enzyme. Besides, the medium composition and culture conditions were optimized for maximum sucrolytic enzyme production. In addition, the properties of the obtained enzyme, as well as its purification, were studied.

## 2. Materials and Methods

### 2.1. Materials

*B. licheniformis* TKU004 was isolated and described in an earlier report [46]. Sucrose, glucose, maltose, and fructose were bought from Katayama Chemical Industries Corporation (Osaka, Japan). 3,5-Dinitrosalicylic acid (DNS), and fructooligosaccharide (FOS) were purchased from Sigma Aldrich (Saint Louis, MO, USA). Rice bran and wheat bran were collected from Miaoli (Miaoli City, Taiwan). Orange, banana, and pomelo were bought from the local markets of Tamshui (New Taipei, Taiwan) and then peeled to prepare orange peel, banana peel, pomelo albedo, and pomelo flavedo. Finally, these materials were dried and ground to powder (particle size smaller than 0.297 mm). All other chemicals were of the highest possible quality.

### 2.2. Sucrolytic Assay

Sucrolytic activity was determined using the DNS method [21] with certain modifications. Briefly, a mixture of 50 µL enzyme and 150 mL sucrose (1%, *w/v*) was incubated at 37 °C for 60 min to allow the hydrolysis of sucrose. Later, 750 µL of DNS reagent was added to the reaction and the obtained mixture was heated at 100 °C for 10 min. Consequently, the absorbance of red color of the mixture was read at 515 nm using an ELISA reader (Bio-Rad, Hercules, CA, USA) and was used to calculate the amount of reducing sugar in the hydrolysis reaction. The amount of enzyme required to release 1 μM of reducing sugar in one minute was described as one unit of sucrolytic activity.

### 2.3. Agro-Byproducts as the Sole Carbon Source for Sucrolytic Enzymes Production

One percent of each carbon source (PAP, PFP, OPP, BPP, RBP, WBP, and sucrose) was added into a 250 mL flask containing 100 mL of basal medium (1% KNO_3_, 0.1% KH_2_PO_4_, and 0.05% MgSO_4_). The bacterial seed was prepared by adding a loopful of pure culture into a 250 mL flask containing 100 mL of nutrient broth and then culturing at 37 °C and 150 rpm for 24 h. The cultivation of *B. licheniformis* TKU004 on different carbon source-containing mediums was initiated by adding 1 mL of the bacterial seed solution (OD 660 nm = 1) to each medium and kept at 37 °C and 150 rpm. One milliliter of culture medium was withdrawn each 24 h and the sucrolytic activity was estimated. Different amounts of PAP (0.25 g, 0.5 g, 1.0 g, 1.5 g, 2.0 g, 2.5 g, and 3 g) were used to explore the optimal PAP concentration for sucrolytic enzyme production. Each experiment was performed in triplicate.

### 2.4. Effect of Other Conditions on Sucrolytic Enzymes Production

To explore the effect of other conditions on sucrolytic enzyme production by *B. licheniformis* TKU004, the one-factor-at-a-time method was used in the order of nitrogen source (KNO_3_, NH_4_NO_3_, (NH_4_)_2_SO_4_, casein, peptone, beef extract powder (BEP), squid pens powder (SPP), shrimp shells powder (SSP), and shrimp heads powder (SHP)), NH_4_NO_3_ concentration (0%, 0.25%, 0.50%, 0.75%, 1%, 1.25%, 1.50%, 1.75%, and 2%), phosphate salts (NaH_2_PO_4_, Na_2_HPO_4_, KH_2_PO_4_, and K_2_HPO_4_), NaH_2_PO_4_ concentration (0%, 0.025%, 0.05%, 0.075%, and 0.1%), yeast extract powder concentration (0%, 0.05%, 0.1%, 0.15%, and 0.2%), pH (3.7, 4.7, 5.7, 6.7, 7.7, and 8.7), temperature (25 °C, 30 °C, 35 °C, 37 °C, and 40 °C), shaking speed (100 rpm, 125 rpm, 150 rpm, 175 rpm, and 200 rpm), and cultivation time (0 h, 12 h, 24 h, 36 h, 48 h, 60 h, and 72 h). The original conditions for the experiments were 2% PAP, 1% KNO_3_, 0.1% KH_2_PO_4_, 0.05% MgSO_4_, pH = 5.7, 37 °C, 100/250 mL, 150 rpm, and 24 h. At a time, only one factor was assessed while other factors were constant and the condition giving the highest sucrolytic activity was chosen for further experiments. Each experiment was performed in triplicate.

### 2.5. Enzyme Purification

To obtain cell-free supernatant, the culture medium of *B. licheniformis* TKU004 was centrifuged (9000× rpm for 30 min, 320 R, Hettich, Tuttlingen, Germany) and then mixed with (NH_4_)_2_SO_4_ (60% *w/v*, 4 °C, overnight). The precipitate was conveniently removed from the mixture by centrifugation (10.000× *g* rpm, 30 min) and dissolved in sodium phosphate buffer (50 mM, pH = 7). The residual (NH_4_)_2_SO_4_ in the crude enzyme solution was removed using a cellulose membrane (CelluSep T2, Interchim, Montluçon, France). The crude enzyme solution was then loaded onto a DEAE sepharose column pre-equilibrated with sodium phosphate buffer (50 mM, pH = 7). A linear gradient of NaCl (0 M–1.0 M) was applied to elute the target enzymes. Fractions exhibiting sucrolytic activity were then pooled and concentrated by lyophilization. Finally, the obtained enzyme was purified by a high-performance liquid chromatography (HPLC, Hitachi Chromaster HPLC system, Hitachi, Tokyo, Japan) system consisting of a KW-802.5 column. The molecular weight (MW) of the obtained enzyme was determined using sodium dodecyl sulfate-polyacrylamide gel electrophoresis (SDS-PAGE) and matrix-assisted laser desorption ionization-time of flight mass spectrometry (MALDI-TOF MS, Bruker Daltonics, Bremen, Germany) methods, as described in early reports [22,47]. The identification of the obtained enzyme was determined by liquid chromatography-tandem mass spectrometry (LC-MS/MS, performed by Mission Biotech, Taipei, Taiwan) [48].

### 2.6. Effect of Temperature and pH

The optimum temperature of sleTKU004 activity was examined over a range of 30 °C to 80 °C. The thermal stability of sleTKU004 was accorded to its residual sucrolytic activity after allowing the sleTKU004 solution to stand at 20 °C, 40 °C, 50 °C, and 60 °C for 0.5 h, 1 h, 2 h, and 4 h. The optimum pH and pH stability of sleTKU004 were explored at the pH range of 3 to 10 using a system buffer (sodium carbonate buffer, pH 9–10; sodium phosphate buffer, pH 6–8; sodium acetate buffer, pH = 5; glycine HCl buffer, pH = 3–4). The pH stability was accorded to the residual sucrolytic activity after allowing the sleTKU004 solution to stand at different pH points for 1 h at 20 °C. Each experiment was performed in triplicate.

### 2.7. Effect of Various Chemicals

Various chemicals including KCl, BaCl_2_, CaCl_2_, CaSO_4_, FeCl_3_, FeCl_2_, CuSO_4_, ZnSO_4_, MgSO_4_, MnSO_4_, ethylenediaminetetraacetic acid (EDTA), Tween 20, Tween 40, Triton X-100, and sodium dodecyl sulfate (SDS) were used to examine their effect on the sucrolytic activity of sleTKU004. KCl, BaCl_2_, CaCl_2_, CaSO_4_, FeCl_3_, FeCl_2_, CuSO_4_, ZnSO_4_, MgSO_4_, MnSO_4_, and EDTA were tested at the final concentration of 1 mM, 5 mM, and 10 mM whereas Tween 20, Tween 40, Triton X-100, and SDS were assessed at 1%, 5%, and 10% concentrations. Each experiment was performed in triplicate.

### 2.8. Substrate Specificity

The specificity of the sleTKU004 enzyme was tested on substrates including sucrose, raffinose, stachyose, FOS, pectin, dialyzed pectin, dextran starch, gum arabic, and 1,3-β-glucan using the conditions of the sucrolytic activity assay (as described above). All the substrates were prepared at a concentration of 1% (*w*/*v*). Each experiment was performed in triplicate. The HPLC analysis of the sucrose, raffinose, stachyose hydrolysis was conducted under the following conditions: NH_2_P-50 4E column; a mobile phase of 75% acetonitrile and 25% water; flowrate of 1.0 mL/min; infrared (IR) detector.

### 2.9. Effect of Sucrose Concentration

The effect of sucrose concentration on the sucrolytic activity of sleTKU004 was examined by mixing sleTKU004 solution with sucrose at different concentrations to gain the final sucrose concentration of 0.2 M to 1.5 M. Sucrolytic activity was then determined following the assay described above. Each experiment was performed in triplicate.

### 2.10. Statistical Analysis

The experiments pertaining to the culture parameters and effects of sucrose concentration on the activity of sleTKU004 were analyzed through one-way analysis of variance (ANOVA) and the post hoc Duncan’s Multiple Range Test. The experiment on the effects of various chemicals on the activity of sleTKU004 was performed using one-way ANOVA with the post hoc Tukey Honestly Significant Difference (HSD). Statistical significance was achieved when *p* < 0.05.

## 3. Results and Discussion

### 3.1. Agro-Byproducts as the Sole Carbon Source for Sucrolytic Enzyme Production

**Figure 1:**
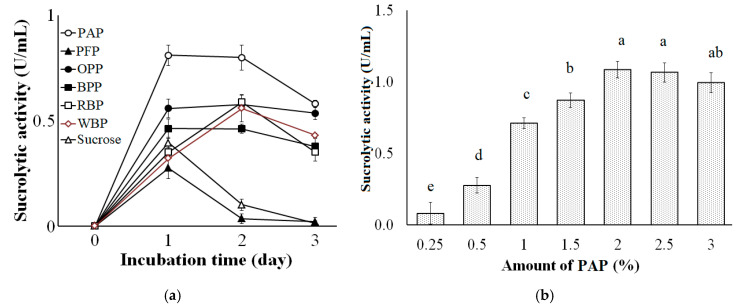
Effect of different carbon sources (**a**) and amount of PAP (**b**) on the sucrolytic enzyme production by *B. licheniformis* TKU004. PAP, pomelo albedo powder; PFP, pomelo flavedo powder; OPP, orange peel powder; BPP, banana peel powder; RBP, rice bran powder; WBP, wheat bran powder. All data points are the mean ± standard deviation. Sucrolytic activity values with different letters (a–e) are significantly different based on Duncan’s Multiple Range Test at the level of 5%.

Various carbon sources were examined for the production of sucrolytic enzymes by *B. licheniformis* TKU004. These included PAP, PFP, OPP, BPP, RBP, WBP, and sucrose. As shown in Figure 1a, the highest sucrolytic activity was observed in the culture supernatant containing PAP as the carbon source (0.81 U/mL on the first day of fermentation), followed by OPP (0.56 U/mL on the 1st day and 0.58 U/mL on the 2nd day of fermentation), RBP (0.59 U/mL on the 2nd day of fermentation), WBP (0.56 U/mL on the 2nd day of fermentation), BPP (0.46 U/mL on the 1st day and 0.46 U/mL on the 2nd day of fermentation), sucrose (0.38 U/mL on the 1st day of fermentation), and PFP (0.28 U/mL on the 1st day of fermentation). Pomelo albedo contains 72.62% carbohydrate, 16.13% moisture, 6.27% protein, 3.41% ash and 1.56% fat [49]. Huang et al., (2014) reported that the carbohydrates of pomelo peel include pectin (35.42%), cellulose (16.50%), hemicellulose (6.86%), lignin (3.16%), and soluble sugar (12.62%) [50]. The high carbohydrate ratio, especially the soluble sugar could make the pomelo albedo an attractive carbon source for microbial fermentation. Currently, bioethanol production is the primary commercial operational process that utilizes pomelo peel waste as a raw material [51,52]. The potential of pomelo albedo in the manufacture of value-added products such as enzymes is not yet fully explored. In this study, among the tested byproducts, pomelo albedo exhibited the highest enzyme activity and was thus chosen as the most suitable carbon source for sucrolytic enzyme production by *B. licheniformis* TKU004.

To examine the effect of PAP concentration on the production of sucrolytic enzymes, the amount of PAP was adjusted in a range of 0.25–3% (*w/v*). As shown in Figure 1B, the increase of PAP concentration from 0.25% to 2% could significantly increase the production of sucrolytic enzymes (from 0.08 U/mL to 1.09 U/mL, respectively). Besides, higher PAP concentration (2%, 2.5%, 3%) did not show any significant improvement in the production of sucrolytic enzymes (1.09 U/mL, 1.07 U/mL, and 0.99 U/mL). Due to the lower cost PAP material and the highest sucrolytic enzyme productivity, 2% PAP was chosen as the most suitable concentration for the production of sucrolytic enzymes by *B. licheniformis* TKU004. From the literature, various substances have been used as suitable carbon sources for producing sucrolytic enzymes, such as sucrose [43], glucose [53], fructose [54], orange peel [55], and pineapple crown [56].

### 3.2. Effect of Nitrogen Sources on Sucrolytic Enzymes Production

**Figure 2:**
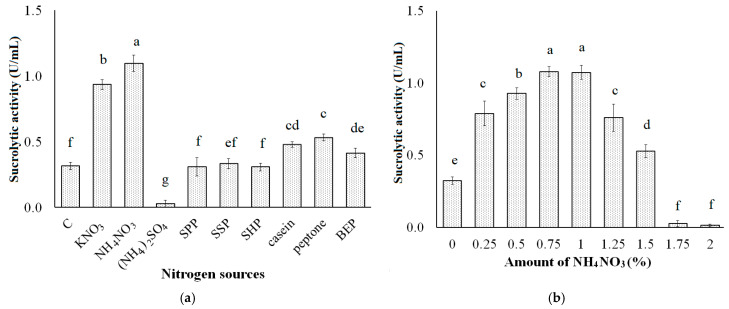
Effect of different nitrogen sources (**a**) and amount of NH_4_NO_3_ (**b**) on the sucrolytic enzyme production by *B. licheniformis* TKU004. C, control; SPP, squid pens powder; SSP, shrimp shells powder; SHP, shrimp heads powder; BEP, beef extract powder. All data points are the mean ± standard deviation. Sucrolytic activity values with different letters (a–g) are significantly different based on Duncan’s Multiple Range Test at the level of 5%.

Pomelo albedo has a low nitrogen content and comprises only 6.27% protein [49]. Thus, supplementing the nitrogen source to a PAP-containing medium may be a necessity. Accordingly, different nitrogen sources such as KNO_3_, NH_4_NO_3_, (NH_4_)_2_SO_4_, casein, peptone, BEP, SPP, SSP, and SHP were supplemented to the PAP-containing medium to assess the most suitable nitrogen source for the production of sucrolytic enzymes by *B. licheniformis* TKU004. As shown in Figure 2a, a higher sucrolytic activity was observed using NH_4_NO_3_ (1.10 U/mL), followed by KNO_3_ (0.94 U/mL), peptone (0.53 U/mL), casein (0.48 U/mL), and BEP (0.41 U/mL). The sucrolytic activity of SSP-, SPP-, and SHP-containing mediums were not significantly different from that of the control medium (0.33 U/mL, 0.31 U/mL, 0.31 U/mL, and 0.31 U/mL respectively), which only contained 2% PAP, 0.1% KH_2_PO_4_, and 0.05% MgSO_4_. Besides, the use of 1% (NH_4_)_2_SO_4_ could potentially inhibit the production of sucrolytic enzymes by *B. licheniformis* TKU004 (0.03 U/mL). These results indicate that NH_4_NO_3_ is the best nitrogen source as a supplement to PAP-containing medium for the production of sucrolytic enzymes by *B. licheniformis* TKU004. There are reports of various organic and inorganic nitrogen sources ideal for high sucrolytic enzyme production such as yeast extract, peptone, urea, NaNO_3_, tryptone, NH_4_Cl, and (NH_4_)_2_HPO_4_ [15].

The effect of NH_4_NO_3_ concentration on sucrolytic enzyme production was studied by adding 0–2% NH_4_NO_3_ into the PAP-containing medium. As shown in Figure 2b, the sucrolytic activity of medium with NH_4_NO_3_ at 0%, 0.25%, 0.50%, 0.75%, 1%, 1.25%, 1.50%, 1.75% and 2% was 0.31 U/mL, 0.79 U/mL, 0.93 U/mL, 1.08 U/mL, 1.07 U/mL, 0.76 U/mL, 0.53 U/mL, 0.03 U/mL, and 0.02 U/mL, respectively. The sucrolytic activity of the medium containing 0.75% NH_4_NO_3_ and 1% NH_4_NO_3_ were not significantly different, indicating that 0.75% NH_4_NO_3_ is the optimum concentration for the production of the sucrolytic enzyme by *B. licheniformis* TKU004.

### 3.3. Effect of Phosphate Salts and Yeast Extract Powder Concentration on Sucrolytic Enzyme Production

**Figure 3:**
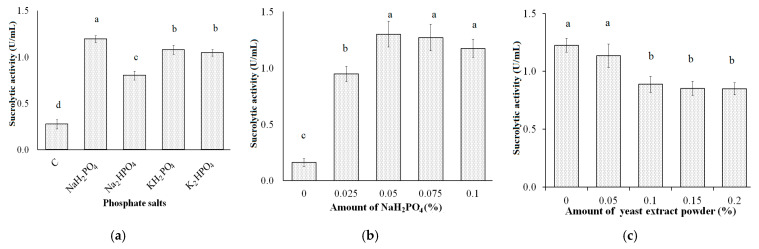
Effect of phosphate salts (**a**), amount of NaH_2_PO_4_ (**b**), and amount of yeast extract powder (**c**) on the sucrolytic enzyme production by *B. licheniformis* TKU004. C, control. All data points are mean ± standard deviation. Sucrolytic activity values with the different letters (a–d) are significantly different based on Duncan’s Multiple Range Test at the level of 5%.

Phosphate salts also affect microbial bio-active productivity [57]. Thus, the effect of some phosphate salts such as NaH_2_PO_4_, Na_2_HPO_4_, KH_2_PO_4_, and K_2_HPO_4_ on the production of sucrolytic enzymes by *B. licheniformis* TKU004 was explored herein. Each of those phosphate salts was added to a medium containing 2% PAP, 0.75% NH_4_NO_3_, and 0.05% MgSO_4_. As shown in Figure 3a, NaH_2_PO_4_ was screened as the most suitable form of phosphate for the optimum sucrolytic activity and was higher than that of the others (1.20 U/mL compared to 0.80 U/mL in Na_2_HPO_4_-containing medium, 1.08 U/mL in KH_2_PO_4_-containing medium, and 1.05 U/mL in K_2_HPO_4_-containing medium). As shown in Figure 3b, the sucrolytic activity of medium with NaH_2_PO_4_ at 0%, 0.025%, 0.05%, 0.075%, and 0.1% was, 0.16 U/mL, 0.95 U/mL, U/mL, 1.30 U/mL, 1.27 U/mL, and 1.17 U/mL (respectively). The sucrolytic activity of the medium containing 0.05% NaH_2_PO_4_ and 0.075% NaH_2_PO_4_ were not significantly different, indicating that 0.05% NaH_2_PO_4_ is the optimum concentration for the production of the sucrolytic enzyme by *B. licheniformis* TKU004.

Various microbial strains, for example, *Pichia* sp. [58], *B. cereus* TA-11 [59], *B. macerans* [60], *Lactobacillus brevis* Mm-6 [61], and *Zymomonas mobilis* CDBB-B 603 [53] need yeast extract to enhance the sucrolytic enzyme productivity. In this study, a range of yeast extract powder from 0% to 0.2% was added to a medium containing 2% PAP, 0.75% NH_4_NO_3_, 0.05% MgSO_4_, and 0.05% NaH_2_PO_4_ to explore its effect on the production of sucrolytic enzymes from *B. licheniformis* TKU004. As shown in Figure 3c, yeast extract powder had a negative effect on the enzyme production and the highest sucrolytic activity was obtained from the medium without the addition of yeast extract powder (1.23 U/mL). In fact, yeast extract powder has a high ratio of proteins (48.52%), and carbohydrates (32.92%) [62]. According to the result, the supplement of yeast extract powder can provide nutrients to the bacteria without the necessity to use sucrose or sucrose-based substances, thereby drastically reducing the production of sucrolytic enzymes.

### 3.4. Effect of Cultural Conditions on Sucrolytic Enzymes Production

**Figure 4:**
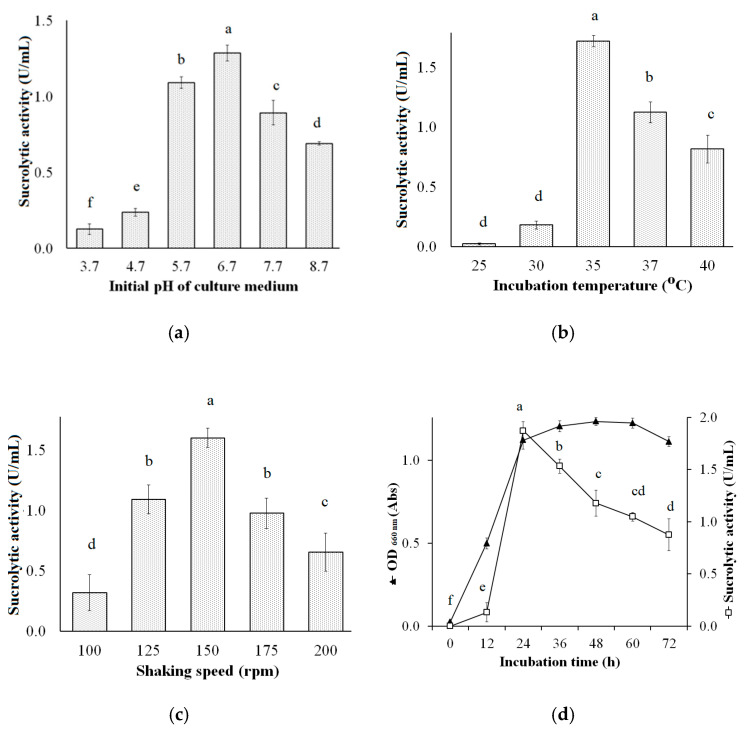
Effect of initial pH (**a**), temperature (**b**), shaking speed (**c**), and incubation time (**d**) on the sucrolytic enzyme production by *B. licheniformis* TKU004. All data points are the mean ± standard deviation. Sucrolytic activity values with different letters (a–f) are significantly different based on Duncan’s Multiple Range Test at the level of 5%.

The optimum sucrolytic enzyme production by *B. licheniformis* TKU004 using pomelo albedo as the sole carbon source was in the medium containing 2% PAP, 0.75% NH_4_NO_3_, 0.05% MgSO_4_, and 0.05% NaH_2_PO_4_. We then explored the effect of the cultural conditions on the sucrolytic enzyme production by *B. licheniformis* TKU004. These included initial pH of the medium (3.7–8.7), cultivation temperature (25–40 °C), shaking speed (100 rpm, 125 rpm, 150 rpm, 175 rpm, and 200 rpm), and cultivation time (0–72 h). The sucrolytic activity produced in media of different pH (Figure 4a) were in the order of pH = 6.7 (1.29 U/mL), pH = 5.7 (1.09 U/mL), pH = 7.7 (0.89 U/mL), pH = 8.7 (0.89 U/mL), pH = 4.7 (0.23 U/mL), and pH = 3.7 (0.13 U/mL); at different temperatures (Figure 4b) were in the order of 35 °C (1.72 U/mL), 37 °C (1.12 U/mL), 40 °C 0.82 U/mL), 30 °C (0.18 U/mL), and 25 °C (0.02 U/mL); and at different shaking speeds (Figure 4c) were in the order of 150 rpm (1.60 U/mL), 125 rpm (1.09 U/mL), 175 rpm (0.97 U/mL), 200 rpm (0.16 U/mL), and 100 rpm (0.15 U/mL). The time course of cell growth and sucrolytic enzyme production of *B. licheniformis* TKU004 under the optimized conditions is shown in Figure 4d. The number of the cells of *B. licheniformis* TKU004 increased quickly at 0–24 h and then entered the stationary phase in the 24–60 h period. The sucrolytic activity of the culture medium also reached its maximum in the early stationary phase (1.87 U/mL at 24 h) and then decreased over time. Likewise, the time course for sucrolytic enzyme production by bacteria was around 12–72 h [15] for example *Streptomyces* sp. ALKC8 (24 h, 0.35 U/mL) [63], *Brevibacterium divaricatum* (12 h) [64], *Lactobacillus brevis* Mm-6 (72 h, 1399 U/mL) [61], and *Arthrobacter* sp.10137 (22.5 h, 26.69 U/mL) [65]. Another study found that the short fermentation period has more advantages than the long one [21]. This suggested that the time course for the production of sucrolytic enzyme by *B. licheniformis* TKU004 was acceptable. From the literature, the optimum medium for the production of sucrolytic enzymes may require the supplement of sugar (sucrose [43], fructose [54], and glucose [53]) and organic nitrogen (peptone [60], malt extract [61], and yeast extract [58]). However, the price of those materials may be a significant obstacle in the production of sucrolytic enzymes. Thus, to reduce the cost of the process of sucrolytic enyzme production, the use of agricultural wastes can be an effective alternative. In this study, we could successfully develop a low-cost and simple medium based on pomelo albedo and optimize the culture conditions for sucrolytic enzyme production by *B. licheniformis* TKU004. The composition of the optimal medium was 2% PAP, 0.75% NH_4_NO_3_, 0.05% MgSO_4_, and 0.05% NaH_2_PO_4_, and the optimal culture conditions were observed to be initial medium pH of 6.7, cultivation temperature of 35 °C, shaking speed of 150 rpm, and cultivation time of 24 h. Accordingly, the highest sucrolytic activity was 1.87 U/mL, 4.79-fold higher than that in original conditions using sucrose as the carbon source.

### 3.5. Purification of the Produced Sucrolytic Enzyme

**Figure 5:**
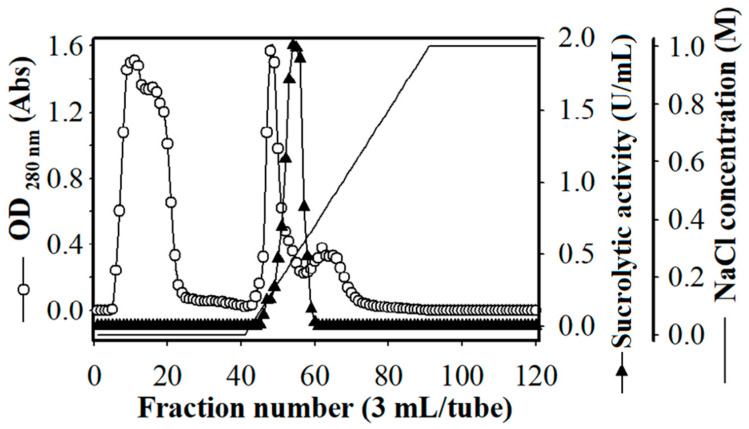
DEAE-Sepharose chromatography profile of the crude sleTKU004 enzyme.

**Figure 6:**
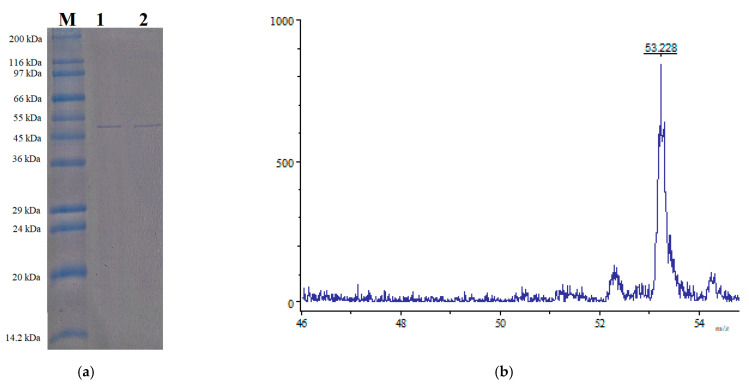
SDS-PAGE (**a**) and MALDI-TOF MS profile (**b**) of the sleTKU004. M, protein markers; 1, with 2-mercaptoethanol (2-ME); 2, without 2-ME.

One-day culture supernatant (0.4 L) was used to purify the produced sucrolytic enzymes under the following condition: DEAE-Sepharose resin, 50 mM sodium phosphate (pH = 7.0), and NaCl gradient from 0 M to 1 M. The fractions showing sucrolytic activity from tube number 45 to 59 (Figure 5) were dialyzed against 50 mM sodium phosphate (pH = 7.0), and concentrated by a freeze-dryer. HPLC with size-exclusion chromatography mode was then used for the purification of the enzyme. After purification, approximately 1.34 mg of *B. licheniformis* TKU004 sucrolytic enzyme (sleTKU004) was obtained. The recovery yield of the obtained enzyme was 1.31% with 11.4-folds of the specific activity (Table 1). As shown in Table 2, the MW of 53 kDa (by SDS-PAGE method) of sleTKU004 is highly similar to sucrase from *B. stearothermophilus* NUB36 (51.519 Da) and levansucrases from *B. licheniformis* RN-01 (52 kDa), *B. licheniformis* 8-37-0-1 (51 kDa), *B. amyloliquefaciens* BH072 (55 kDa), and *B. amyloliquefaciens* KK9 (52974 Da). In general, the sucrolytic enzymes from the *Bacillus* genus have an MW range of 14–105 kDa (Table 2). The non-reducing SDS-PAGE analysis of sleTKU004 also revealed a single band at 53 kDa (Figure 6a), indicating that the enzyme is a monomer in its native form. Other reports show that the sucrose hydrolytic enzymes from the *Bacillus* genus could be monomers [66] or dimers [67]. The native form of sleTKU004 was also analyzed by MALDI-TOF MS and a peak was observed at 53,228 Da (Figure 6b).

### 3.6. Identification of sleTKU004 by LC-MS/MS Analysis

To identify sleTKU004 that appeared as a prominent 53-kDa band via SDS-PAGE, the band was excised and analyzed after tryptic digestion. The excised band from the gel was subjected to electrospray tandem mass spectrometry analysis. The fragment spectra were subjected to an NCBI non-redundant protein database search. As shown in Table 3, the spectra of sleTKU004 matched nine tryptic peptides that were identical to the levansucrase (sacB) from *B. subtilis* subsp. *subtilis* str. 168 with 30% sequence coverage. The peptide sequences indicate that sleTKU004 belongs to the family 68 glycosyl hydrosylase based on the amino acid sequence similarity of the cited GH-68 enzymes from *B. subtilis*. Besides, the MW of sleTKU004 (53 kDa) was also similar to that of levansucrase from *B. subtilis* subsp. *subtilis* str. 16 (52.938 Da).

### 3.7. Effects of Temperature and pH on the Activity and Stability of sleTKU004

**Figure 7:**
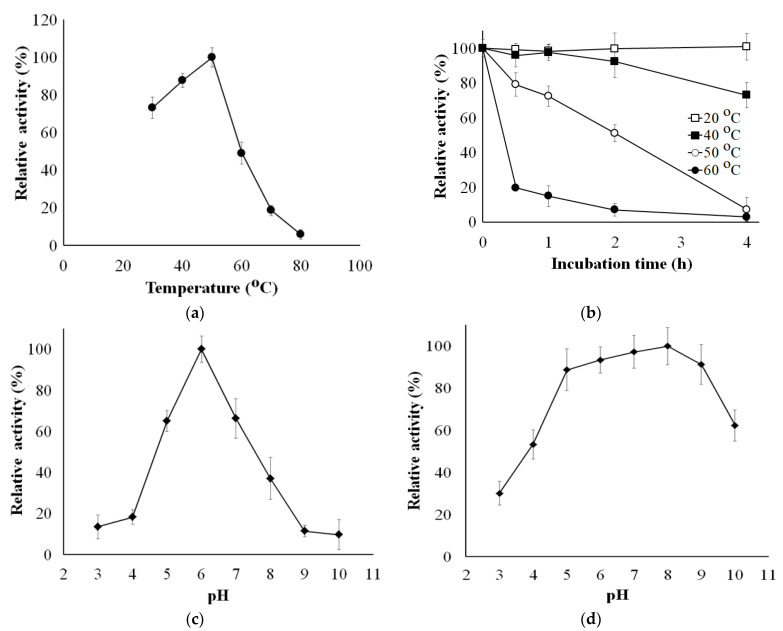
Effects of Temperature and pH on the activity and stability of sleTKU004. (**a**), optimum temperature; (**b**), thermal stability; (**c**), optimum pH; (**d**), pH stability. All data points are the mean ± standard deviation.

The optimum temperature for sucrolytic activity of sleTKU004 was examined in a temperature range of 30–80 °C. The sucrolytic activity of the enzyme increased with a rise in temperature from 30 °C to 50 °C, and then sharply decreased at higher temperatures (Figure 7a). The optimum temperature for sucrolytic activity of sleTKU004 was 50 °C. Likewise, sucrolytic enzymes from *Bacillus* genus have also been found to work best in the temperature range of 30 °C to 60 °C. To explore the thermal stability of sleTKU004, the enzyme was treated at 20 °C, 40 °C, 50 °C, and 60 °C for 0.5–4 h. The result showed that the sucrolytic activity of sleTKU004 could be retained to 100% at 20 °C in 4 h, 92% at 40 °C in 3 h, and 73% at 40 °C in 4 h (Figure 7b). At higher temperatures, the half-life of sleTKU004 was estimated to be around 2 h (at 50 °C), and 0.3 h (at 60 °C). The pH activity profile sleTKU004 is shown in Figure 7c,d. The optimum enzyme activity was observed at pH = 6 and it stable at the pH range of pH = 5–9. Likewise, many sucrolytic enzymes from other *Bacilli* expressed the highest activity at pH = 6 (Table 2).

### 3.8. Effect of Various Chemicals on the Activity of sleTKU004

**Figure 8:**
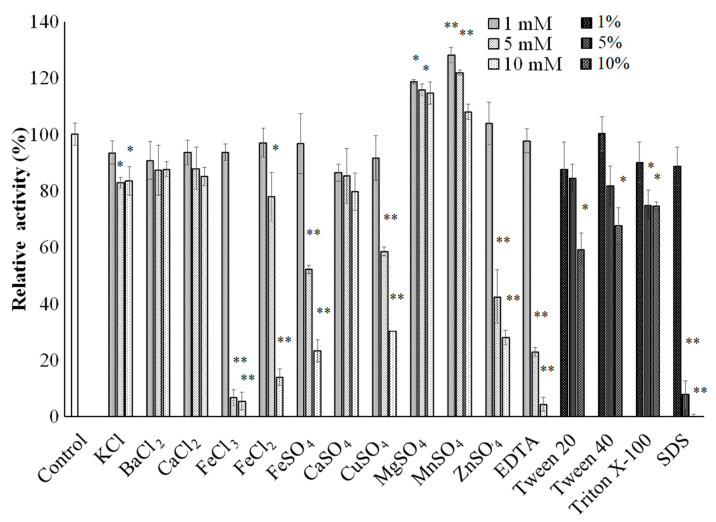
Effect of various chemicals on the activity of sleTKU004. All data points are the mean ± standard deviation. All data points are the mean ± standard deviation. Asterisks were used to highlight the statistical differences between control and treatment groups using the Tukey HSD Test. *, and ** indicate statistical significance at *p* < 0.05 and *p* < 0.01, respectively.

Generally, metal ions have a great influence on protein folding and catalytic processes [58]. As shown in Figure 8, some metal salts such as KCl, BaCl_2_, CaCl_2_, and CaSO_4_ had no significant effect or only slightly inhibited the sucrolytic activity of sleTKU004. At 1 mM, FeCl_3_, FeCl_2_, FeSO_4_, CuSO_4_, ZnSO_4,_ and EDTA did not affect sleTKU004, however, at higher concentrations (5 mM, and 10 mM), they could inhibit the sucrolytic activity of the enzyme. On the contrary, MgSO_4_, and MnSO_4_ in the concentration range of 1 mM–10 mM slightly promoted the sucrolytic activity of sleTKU004. Tween 20, Tween 40, Triton X-100, and SDS were also tested for their effect on sleTKU004 sucrolytic activity. A slight inhibiting effect was found on tween 20 (10%), tween 40 (10%), and triton X-100 (5% and 10%). At 1%, SDS did not affect sleTKU004, however, at higher concentrations (5% and 10%) it completely inhibited the enzyme activity.

### 3.9. Substrate Specificity of sleTKU004

Substrate specificity sleTKU004 is summarized in Table 4. In the case of disaccharide and oligosaccharide substrates, sleTKU004 expressed the greatest activity in the order of sucrose (100%) > raffinose (85.74%) > stachyose (73.60%) > FOS (19.15%). No activity was observed against maltose and sucralose. In the case of polysaccharide substrates, sleTKU004 seemingly showed high pectinolytic activity and expressed a relative activity of 81.38% on pectin. Surprisingly, a significant amount of residual sucrose was found in the pectin substrate and was the primary contributor to the production of reducing sugar which was then used to calculate the enzyme’s relative activity (data not shown). Thus, 2-days dialyzed pectin was used to test the hydrolysis activity of sleTKU004, and the enzyme only expressed 15.26% relative activity on this type of pectin. Likewise, the relative activity on dextran was 16.94%. These results indicate that sleTKU004 may have a slight pectinolytic, and dextranolytic activity. Besides, the activity of sleTKU004 was insignificant on starch (8.82%), gum arabic (9.96%), and 1,3-β-glucan (3.59%).

**Figure 9:**
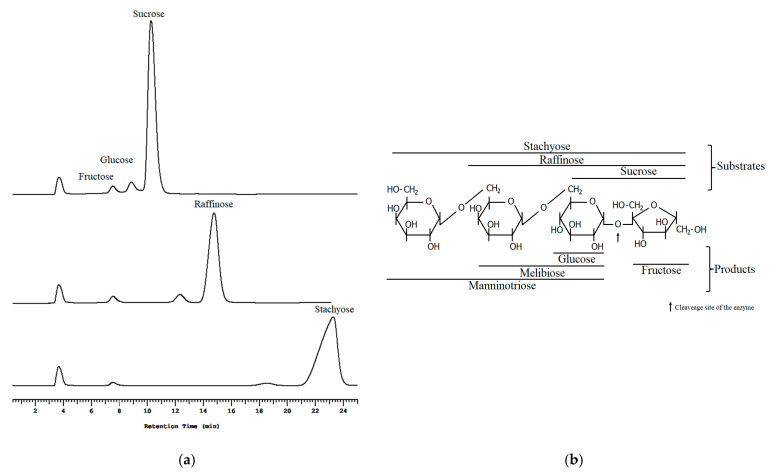
HPLC profile (**a**) and schematic diagram (**b**) of the sucrose, raffinose, and stachyose hydrolysis catalyzed by sleTKU004.

The mechanism of high hydrolytic activity of sleTKU004 on sucrose, raffinose, and stachyose was further analyzed by HPLC. The results showed that fructose was one of the major products of the hydrolysis, indicating that sleTKU004 strictly attacked the glycoside linkage between glucose and fructose molecules of the sucrose unit (Figure 9). The other products of sucrose, raffinose, and stachyose hydrolysis were found to be glucose, melibiose, and manninotriose.

### 3.10. Effect of Sucrose Concentration and Kinetic Characterization

**Figure 10:**
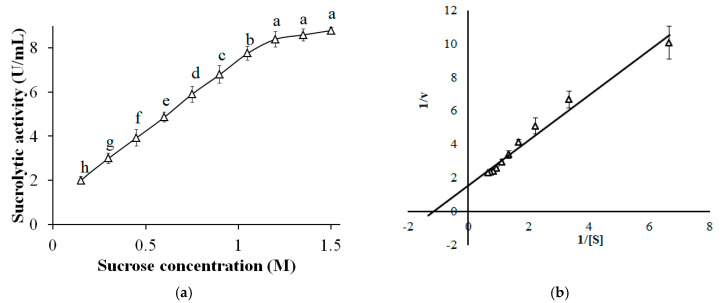
The effect of sucrose concentration on the activity of sleTKU004 (**a**) and the Lineweaver Burk plot (**b**). All data points are the mean ± standard deviation. Sucrolytic activity values with different letters (a–h) are significantly different based on Duncan’s Multiple Range Test at the level of 5%.

The effect of sucrose concentration on the activity of sleTKU004 was shown in Figure 10a. With an increase in sucrose concentration from 0.15 M to 1.2 M, there was a gradual increase in the sucrolytic activity of sleTKU004 (1.98–8.39 U/mL, respectively). Higher sucrose concentrations (1.2–1.5 M) maintained the high sucrose hydrolytic activity (8.39–8.79 U/mL). It was shown that the enzymatic method is preferable in the production of high-quality invert syrups compared with the acidic method. However, high sucrose concentration, which is regarded as one of the inhibitors that block the preparation of inverted sugar [2], could be a significant obstacle for the application of the enzymatic method. For this reason, candidates producing sucrolytic enzymes, which are tolerant to high-concentration sucrose, are being explored for the production of high-quality invert sugar syrups [2]. Together, the result indicates that sleTKU004 could be a potential candidate for the inverted sugar preparation.

*K_m_* and *V_max_* of sleTKU004 were estimated to be 1.16 M and 5.99 µmol/min based on a Lineweaver-Burk plot (Figure 10b). *K_m_* value of sleTKU004 was higher than that of sucrolytic enzymes from other *Bacilli* such as *B. methylotrophicus* SK 21.002 (117.2 mM) [77], *B. amyloliquefacien* BH072 (0.71 mM) [73], *B. cereus* TA-11 (370 mM) [59], and *B. subtilis* (33.96 g/L) [69]. A high *K_m_* value indicates that high concentration of sucrose must be present to saturate sleTKU004. This suggests that the affinity of sleTKU004 towards sucrose is low [59].

## 4. Conclusions

The current study aimed to establish bioprocessing of agro-byproducts for sucrolytic enzyme production by *B. licheniformis* TKU004. A medium containing PAP as the sole carbon source was found to be the most suitable for sucrolytic enzyme production. Then, the culture condition for PAP-containing mediums was successfully optimized with a sucrolytic activity 4.79-fold higher than that from original conditions. A 53 kDa sucrolytic enzyme (sleTKU004) was isolated from the PAP culture supernatant and purified and was then determined to belong to the GH 68 family. Furthermore, the characteristics of sleTKU004 were also determined. Finally, sleTKU004 showed strong sucrose tolerance and presented the highest hydrolytic activity at sucrose concentration of 1.2–1.5 M, indicating that sleTKU004 may be a good candidate for industrial applications requiring sucrolytic enzymes.

## Figures and Tables

**Table 1:** A summary of the purification of sleTKU004 from *B. licheniformis* TKU004.

Step	Total Protein (mg)	Total Activity (U)	Specific Activity (U/mg)	Recovery (%)	Purification (Fold)
Cultural supernatant	1170.91	582.31	0.497	100.00	1.00
(NH_4_)_2_SO_4_ precipitation	359.71	297.74	0.827	51.13	1.66
Ion-exchange chromatography	79.98	199.47	2.494	34.26	5.02
Size-exclusion chromatography	1.34	7.63	5.670	1.31	11.40

**Table 2:** A comparison of sleTKU004 with sucrolytic enzyme produced by other *Bacillus* strains.

Enzyme/Strain	Opt. Temp.	Opt. pH	Carbon Source	MW	Ref
Levansucrase *B. licheniformis* TKU004	50 °C	6	PAP	53 kDa	This study
*β*-d-fructofuranosidase *B. subtilis* LYN12	30–60 °C	4–8	wheat bran and molasses	66 kDa and 64,512.31 Da ^1^	[68]
Levansucrase *B. licheniformis* RN-01	50 °C	6	peptone	52 kDa	[42]
Levansucrase *B. licheniformis* 8-37-0-1	45–50 °C	6	sucrose	51 kDa	[43]
Fructan sucrase *B. subtilis* ZW019	50	5.6	tryptone	58 kDa	[69]
Sucrase and levansucrase *B. subtilis* Marburg				40 kDa	[70]
Invertase *Bacillus* sp. HJ14	30–32.5 °C	8		58 kDa	[71]
Levanase *B. subtilis*			sucrose	73 kDa	[72]
Levansucrase *B. amyloliquefaciens* BH072	40 °C	6	sucrose and corn starch	55 kDa	[73]
Invertase *B. cereus* TA-11	50 °C	7	sucrose	23 kDa and 26 kDa ^2^	[59]
Sucrase *B. stearothermophilus* NUB36	55 °C			51,519 Da ^3^ 105,000 Da ^2^	[66]
*α*-glucosidase *B. subtilis*				66 kDa	[74]
levansucrase *B. amyloliquefaciens* KK9				52,974 Da ^3^	[75]
levansucrase *B. subtilis* NRC16	45 °C	8.2	starch	14 kDa	[76]
Levansucrase *B. methylotrophicus* SK 21.002	40 °C	6.5	sucrose	60 kDa	[77]
Levansucrase *B. licheniformis* ANT 179	60 °C	6	sugarcane juice	25 kDa	[44]
Levansucrase *Bacillus* sp.	60 °C	6	starch	56 kDa	[78]

^1^, LC/MS; ^2^, gel filtration; ^3^, deduced polypeptide.

**Table 3:** Identification of sleTKU004 by LC-MS/MS Analysis.

Matched Peptide Sequence	Identified Protein and Coverage Rate	Mass and pI	Strain
^39^ETYGISHITR^48^ ^63^YQVPEFDSSTIK^74^ ^115^NADDTSIYMFYQKVGETSIDSWK^137^ ^145^DSDKFDANDSILK^157^ ^177^LFYTDFSGK^185^ ^216^SIFDGDGKTYQNVQQFIDEGNYSSGDNHTLR^246^ ^326^KVMKPLIASNTVTDEIER^343^ ^421^GNNVVITSYMTNR^433^ ^461^DSILEQGQLTVNK^473^	Levansucrase 30%	52,938 Da 6.18	*B. subtilis* subsp. *subtilis* strain 168

Superscripts (^39^ and ^48^; ^63^ and ^74^; ^115^ and ^137^; ^145^ and ^157^; ^177^ and ^185^; ^216^ and ^246^; ^326^ and ^343^; ^421^ and ^433^; ^461^ and ^473^) indicate regions in the protein sequence of the levansucrase from *B. subtilis* subsp. *subtilis* strain 168.

**Table 4:** Substrate specificity of sleTKU004.

Substrate	Relative Activity (%)
Sucrose	100.00 ± 4.44
Raffinose	85.74 ± 3.45
Stachyose	73.60 ± 5.39
FOS	19.15 ± 2.47
Starch	8.82 ± 1.93
Pectin	81.38 ± 2.60
Dialyzed pectin	15.26 ± 3.28
Dextran	16.94 ± 5.04
Gum arabic	9.96 ± 1.65
1,3-*β*-glucan	3.59 ± 2.24
Maltose	N.A.
Sucralose	N.A.

N.A.: no activity. All data points are the mean ± standard deviation.

## Data Availability

The data presented in this study are available on request from the corresponding author.

## References

[1] Xu W., Ni D., Zhang W., Guang C., Zhang T., Mu W. (2019). Recent advances in levansucrase and inulosucrase: Evolution, characteristics, and application. Crit. Rev. Food Sci. Nutr..

[2] Zhou G., Peng C., Liu X., Chang F., Xiao Y., Liu J., Fang Z. (2020). Identification and immobilization of an invertase with high specific activity and sucrose tolerance ability of *Gongronella* sp. w5 for high fructose syrup preparation. Front. Microbiol..

[3] Hill A., Karboune S., Narwani T.J., de Brevern A.G. (2020). Investigating the product profiles and structural relationships of new levansucrases with conventional and non-conventional substrates. Int. J. Mol. Sci..

[4] Koirala P., Maina N.H., Nihtilä H., Katina K., Coda R. (2021). Brewers’ spent grain as substrate for dextran biosynthesis by *Leuconostoc pseudomesenteroides* DSM20193 and *Weissella confusa* A16. Microb Cell Fact..

[5] Li X., Wang X., Meng X., Dijkhuizen L., Liu W. (2020). Structures, physico-chemical properties, production and (potential) applications of sucrose-derived α-d-glucans synthesized by glucansucrases. Carbohydr. Polym..

[6] Trollope K.M., van Wyk N., Kotjomela M.A., Volschenk H. (2015). Sequence and structure-based prediction of fructosyltransferase activity for functional subclassification of fungal GH32 enzymes. FEBS J..

[7] Le Roy K., Lammens W., Verhaest M., de Coninck B., Rabijns A., van Laere A., van den Ende W. (2007). Unraveling the difference between invertases and fructan exohydrolases: A single amino acid (Asp-239) substitution transforms Arabidopsis cell wall invertase1 into a fructan 1-exohydrolase. Plant. Physiol..

[8] Ackerman D.L., Craft K.M., Townsend S.D. (2017). Infant food applications of complex carbohydrates: Structure, synthesis, and function. Carbohydr. Res..

[9] González-Garcinuño A., Tabernero A., Domínguez A., Galán M.A., del Valle E.M.M. (2018). Levan and levansucrases: Polymer, enzyme, micro-organisms and biomedical applications. Biocatal. Biotransform..

[10] Taskin M., Ortucu S., Unver Y., Canli O. (2016). Invertase production and molasses decolourization by cold-adapted filamentous fungus *Cladosporium herbarum* ER-25 in non-sterile molasses medium. Process. Saf. Environ. Protect. Instit. Chem. Eng..

[11] Hoffmann J.J., Hövels M., Kosciow K., Deppenmeier U. (2020). Synthesis of the alternative sweetener 5-ketofructose from sucrose by fructose dehydrogenase and invertase producing *Gluconobacter* strains. J. Biotechnol..

[12] Rasbold L.M., Heinen P.R., da Conceição Silva J.L., Simão R.d.C.G., Kadowaki M.K., Maller A. (2021). *Cunninghamella echinulata* PA3S12MM invertase: Biochemical characterization of a promiscuous enzyme. J. Food Biochem..

[13] Contesini F.J., de Alencar Figueira J., Kawaguti H.Y., de Barros Fernandes P.C., de Oliveira Carvalho P., da Graça Nascimento M., Sato H.H. (2013). Potential applications of carbohydrases immobilization in the food industry. Int. J. Mol. Sci..

[14] Manoochehri H., Hosseini N.F., Saidijam M., Taheri M., Rezaee H., Nouri F. (2020). A review on invertase: Its potentials and applications. Biocatal. Agric. Biotechnol..

[15] Lincoln L., More S.S. (2017). Bacterial invertases: Occurrence, production, biochemical characterization, and significance of transfructosylation. J. Basic Microbiol..

[16] Kulshrestha S., Tyagi P., Yadavilli S. (2013). Invertase and its application—A brief review. J. Pharm. Res..

[17] Mariz B.P., Carvalho S., Batalha I.L., Pina A.S. (2021). Artificial enzymes bringing together computational design and directed evolution. Org. Biomol. Chem..

[18] Mehta P.K., Sehgal S., Husain Q., Ullah M. (2019). Microbial enzymes in food processing. Biocatalysis.

[19] Wang C.H., Doan C.T., Nguyen V.B., Nguyen A.D., Wang S.L. (2019). Reclamation of fishery processing waste: A mini-review. Molecules.

[20] Doan C.T., Tran T.N., Nguyen V.B., Vo T.P.K., Nguyen A.D., Wang S.L. (2019). Chitin extraction from shrimp waste by liquid fermentation using an alkaline protease-producing strain, *Brevibacillus parabrevis*. Int. J. Biol. Macromol..

[21] Tran T.N., Doan C.T., Wang S.-L. (2021). Conversion of wheat bran to xylanases and dye adsorbent by *Streptomyces thermocarboxydus*. Polymers.

[22] Doan C.T., Tran T.N., Nguyen V.B., Nguyen A.D., Wang S.L. (2020). Utilization of seafood processing by-products for production of proteases by *Paenibacillus* sp. TKU052 and their application in biopeptides’ preparation. Mar. Drugs.

[23] Doan C.T., Chen C.L., Nguyen V.B., Tran T.N., Nguyen A.D., Wang S.L. (2021). Conversion of wheat bran to pectinases by *Bacillus amyloliquefaciens* and its applications on hydrolyzing banana peels for prebiotics production. Polymers.

[24] Hammami A., Bayoudh A., Abdelhedi O., Nasri M. (2018). Low-cost culture medium for the production of proteases by *Bacillus mojavensis* SA and their potential use for the preparation of antioxidant protein hydrolysate from meat sausage by-products. Ann. Microbiol..

[25] Walia A., Guleria S., Mehta P., Chauhan A., Parkash J. (2017). Microbial xylanases and their industrial application in pulp and paper biobleaching: A review. 3 Biotech..

[26] Doan C.T., Tran T.N., Nguyen V.B., Tran T.D., Nguyen A.D., Wang S.L. (2020). Bioprocessing of squid pens waste into chitosanase by *Paenibacillus* sp. TKU047 and its application in low-molecular weight chitosan oligosaccharides production. Polymers.

[27] Wang C.L., Su J.W., Liang T.W., Nguyen A.D., Wang S.L. (2014). Production, purification and characterization of a chitosanase from *Bacillus cereus*. Res. Chem. Intermed..

[28] Tran T.N., Doan C.T., Nguyen M.T., Nguyen V.B., Vo T.P.K., Nguyen A.D., Wang S.L. (2019). An exochitinase with *N*-acetyl-β-glucosaminidase-like activity from shrimp head conversion by *Streptomyces speibonae* and its application in hydrolyzing β-chitin powder to produce *N*-acetyl-D-glucosamine. Polymers.

[29] Tran T.N., Doan C.T., Nguyen V.B., Nguyen A.D., Wang S.L. (2019). The isolation of chitinase from *Streptomyces thermocarboxydus* and its application in the preparation of chitin oligomers. Res. Chem. Intermed..

[30] Biz A., Finkler A.T.J., Pitol L.O., Medina B.S., Krieger N., Mitchell D.A. (2016). Production of pectinases by solid-state fermentation of a mixture of citrus waste and sugarcane bagasse in a pilot-scale packed-bed bioreactor. Biochem. Eng. J..

[31] Ravindran R., Hassan S.S., Williams G.A., Jaiswal A.K. (2018). A Review on bioconversion of agro-industrial wastes to industrially important enzymes. Bioengineering.

[32] Ohara A., de Castro R.J.S., Nisshde T.G., Dias F.F.G., Bagagli M.P., Sato H.H. (2015). Invertase production by *Asspergillus niger* under solid state fermentation: Focus on physical-chemical parameters, synergistic and antagonistic effects using agro-industrial wastes. Biocatal. Agric. Biotechnol..

[33] Clements L.D., Miller B.S., Streips U.N. (2002). Comparative growth analysis of the facultative anaerobes *Bacillus subtilis, Bacillus licheniformis*, and *Escherichia coli*. Syst. Appl. Microbiol..

[34] Pham J.V., Yilma M.A., Feliz A., Majid M.T., Maffetone N., Walker J.R., Kim E., Cho H.J., Reynolds J.M., Song M.C. (2019). A review of the microbial production of bioactive natural products and biologics. Front. Microbiol..

[35] Deng W., Dong X.F., Tong J.M., Zhang Q. (2012). The probiotic *Bacillus licheniformis* ameliorates heat stress-induced impairment of egg production, gut morphology, and intestinal mucosal immunity in laying hens. Poult. Sci..

[36] Sellami-Kamoun A., Haddar A., Ne H.A., Ghorbel-Frikha B., Kanoun S., Nasri M. (2008). Stability of thermostable alkaline protease from *Bacillus licheniformis* RP1 in commercial solid laundry detergent formulations. Microbiol. Res..

[37] Muras A., Romero M., Mayer C., Otero A. (2021). Biotechnological applications of *Bacillus licheniformis*. Crit. Rev. Biotechnol..

[38] Schallmey M., Singh A., Ward O.P. (2004). Developments in the use of *Bacillus* species for industrial production. Can. J. Microbiol..

[39] Doan C.T., Tran T.N., Nguyen M.T., Nguyen V.B., Nguyen A.D., Wang S.L. (2019). Anti-α-glucosidase activity by a protease from *Bacillus licheniformis*. Molecules.

[40] Fincan S.A., Özdemir S., Karakaya A., Enez B., Mustafov S.D., Ulutaş M.S., Şen F. (2021). Purification and characterization of thermostable α-amylase produced from *Bacillus licheniformis* So-B3 and its potential in hydrolyzing raw starch. Life Sci..

[41] De Boer A.S., Priest F., Diderichsen B. (1994). On the industrial use of *Bacillus licheniformis: A* review. Appl. Microbiol. Biotechnol..

[42] Nakapong S., Pichyangkura R., Ito K., Iizuka M., Pongsawasdi P. (2013). High expression level of levansucrase from *Bacillus licheniformis* RN-01 and synthesis of levan nanoparticles. Int. J. Biol. Macromol..

[43] Lu L., Fu F., Zhao R., Jin L., He C., Xu L., Xiao M. (2014). A recombinant levansucrase from *Bacillus licheniformis* 8-37-0-1 catalyzes versatile transfructosylation reactions. Process. Biochem..

[44] Xavier J.R., Ramana K.V. (2017). Optimization of levan production by cold-active *Bacillus licheniformis* ANT 179 and fructooligosaccharide synthesis by its levansucrase. Appl. Biochem. Biotechnol..

[45] Parrado J., Rodriguez-Morgado B., Tejada M., Hernandez T., Garcia C. (2014). Proteomic analysis of enzyme production by *Bacillus licheniformis* using different feather wastes as the sole fermentation media. Enzyme Microb. Technol..

[46] Wang S.L., Kao T.Y., Wang C.L., Yen Y.H., Chern M.K., Chen Y.H. (2006). A solvent stable metalloprotease produced by *Bacillus* sp. TKU004 and its application in the deproteinization of squid pen for β-chitin preparation. Enzym. Microb. Technol..

[47] Tran T.N., Doan C.T., Nguyen V.B., Nguyen A.D., Wang S.L. (2019). Anti-oxidant and anti-diabetes potential of water-soluble chitosan–glucose derivatives produced by Maillard reaction. Polymers.

[48] Liang T.-W., Lo B.-C., Wang S.-L. (2015). Chitinolytic bacteria-assisted conversion of squid pen and its effect on dyes and pigments adsorption. Mar. Drugs.

[49] Zain N.F.M., Yusop S.M., Ahmad I. (2014). Preparation and characterization of cellulose and nanocellulose from pomelo (*Citrus grandis*) albedo. J. Nutr. Food Sci..

[50] Huang R., Cao M., Guo H., Qi W., Su R., He Z. (2014). Enhanced ethanol production from pomelo peel waste by integrated hydrothermal treatment, multienzyme formulation, and fed-batch operation. J. Agric. Food Chem..

[51] Tocmo R., Pena-Fronteras J., Calumba K.F., Mendoza M., Johnson J.J. (2020). Valorization of pomelo (*Citrus grandis* Osbeck) peel: A review of current utilization, phytochemistry, bioactivities, and mechanisms of action. Compr. Rev. Food Sci. Food Saf..

[52] Multari S., Guzzon R., Caruso M., Licciardello C., Martens S. (2021). Alcoholic fermentation of citrus flavedo and albedo with pure and mixed yeast strains: Physicochemical characteristics and phytochemical profiles. LWT.

[53] Bahena J.M.V., Estrada J.V., Hernandez J.A.S., Lopez J.O. (2006). Expression and improved production of the soluble extracellular invertase from *Zymomonas mobilisin* in *Escherichia coli*. Enzyme Microb. Technol..

[54] Warchol M., Perrin S., Grill J.P., Schneider F. (2002). Characterization of a purified β-fructofuranosidase from *Bifidobacterium infantis* ATCC 15697. Lett. Appl. Microbiol..

[55] Nehad E.A., Atalla S.M. (2020). Production and immobilization of invertase from *Penicillium* sp. using orange peel waste as substrate. Egypt Pharmaceut. J..

[56] Do Nascimento G.C., Batista R.D., Santos C.C.A.D.A., da Silva E.M., de Paula F.C., Mendes D.B., de Oliveira D.P., de Almeida A.F. (2019). *β*-Fructofuranosidase and *β*-D-Fructosyltransferase from new *Aspergillus carbonarius* PC-4 strain isolated from canned peach syrup: Effect of carbon and nitrogen sources on enzyme production. Sci. World J..

[57] Nguyen V.B., Nguyen D.N., Nguyen A.D., Ngo V.A., Ton T.Q., Doan C.T., Pham T.P., Tran T.P.H., Wang S.-L. (2020). Utilization of crab waste for cost-effective bioproduction of prodigiosin. Mar. Drugs.

[58] Ghasemi Y., Mohkam M., Ghasemian A., Rasoul-Amini S. (2014). Experimental design of medium optimization for invertase production by *Pichia* sp.. J. Food Sci. Technol..

[59] Yoon M., Choi W., Kwon S., Yi S., Lee D., Lee J. (2007). Purification and properties of intracellulase invertase from alkalophilic and thermophilic *Bacillus cereus* TA-11. J. Appl. Biol. Chem..

[60] Ahmed S.A. (2008). Invertase production by *Bacillus macerans* immobilized on calcium alginate beads. J. Appl. Sci. Res..

[61] Awad G.E.A., Amer H., Gammal E.W.E., Helmy W.A. (2013). Production optimization of invertase by *Lactobacillus brevis* Mm-6 and its immobilization on alginate beads. Carbohydr. Polym..

[62] Aracri F.M., Cavalcanti R.M.F., Guimaraes L.H.S. (2019). Extracellular tannase from *Aspergillus ochraceus*: Influence of the culture conditions on biofilm formation, enzyme production, and application. J. Microbiol. Biotechnol..

[63] Kaur N., Sharma A.D. (2005). Production, optimization and characterization of extracellular invertase by an actinomycete strain. J. Sci. Ind. Res..

[64] Yamamoto K., Kitamoto Y., Ohata N., Isshiki S. (1986). Purification and properties of invertase from a glutamate-producing bacterium. J. Ferment. Technol..

[65] Xu Z.W., Li Y.Q., Wang Y.H., Yang B. (2009). Production of β-fructofuranosidase by *Arthrobacter* sp. and its application in the modification of stevioside and rebaudioside A. Food Technol. Biotechnol..

[66] Li Y., Ferenci T. (1996). The *Bacillus stearothermophilus* NUB36 surA gene encodes a thermophilic sucrase related to *Bacillus subtilis* SacA. Microbiology.

[67] Chambert R., Rain-Guion M.C., Petit-Glatron M.F. (1992). Readthrough of the *Bacillus subtilis* stop codon produces an extended enzyme displaying a higher polymerase activity. Biochim. Biophys. Acta.

[68] Lincon L., More S.S., Reddy S.V. (2018). Purification and biochemical characterization of β-d-fructofuranosidase from *Bacillus subtilis* LYN12. J. Food Biochem..

[69] Wang J., Xiao H., Zhao F., Zhao B., Xu M., Zhou Z., Han Y. (2021). A fructan sucrase secreted extracellular and purified in one-step by gram-positive enhancer matrix particles. Processes.

[70] Pascal M., Kunst F., Lepesant J.A., Dedonder R. (1971). Characterization of two sucrase activities in *Bacillus subtilis* Marburg. Biochimie.

[71] Zhou J., He L., Gao Y., Han N., Zhang R., Wu Q., Li J., Tang X., Xu B., Ding J. (2016). Characterization of a novel low-temperature-active, alkaline and sucrose-tolerant invertase. Sci. Rep..

[72] Martin I., Débarbouillé M., Ferrari E., Klier A., Rapoport G. (1987). Characterization of the levanase gene of *Bacillus subtilis* which shows homology to yeast invertase. Mol. Gen. Genet..

[73] Mu D., Zhou Y., Wu X., Montalban-Lopez M., Wang L., Li X., Zheng Z. (2021). Secretion of *Bacillus amyloliquefaciens* levansucrase from *Bacillus subtilis* and its application in the enzymatic synthesis of levan. ACS Food Sci. Technol..

[74] Schönert S., Buder T., Dahl M.K. (1998). Identification and enzymatic characterization of the maltose-inducible alpha-glucosidase MalL (sucrase-isomaltase-maltase) of *Bacillus subtilis*. J. Bacteriol..

[75] Phengnoi P., Charoenwongpaiboon T., Wangpaiboon K., Klaewkla M., Nakapong S., Visessanguan W., Ito K., Pichyangkura R., Kuttiyawong K. (2020). Levansucrase from *Bacillus amyloliquefaciens* KK9 and its Y237S variant producing the high bioactive levan-type fructooligosaccharides. Biomolecules.

[76] Salama B.M., Helmy W.A., Ragab T.I.M., Ali M.M., Taie H.A.A., Esawy M.A. (2019). Characterization of a new efficient low molecular weight *Bacillus subtilis* NRC 16 levansucrase and its levan. J. Basic Microbiol..

[77] Li R., Zhang T., Jiang B., Mu W., Miao M. (2015). Purification and characterization of an intracellular levansucrase derived from *Bacillus methylotrophicus* SK 21.002. Biotechnol. Appl. Biochem..

[78] Ammar Y.B., Matsubara T., Ito K., Iizuka M., Limpaseni T., Pongsawasdi P., Minamiura N. (2002). Characterization of a thermostable levansucrase from *Bacillus* sp. TH4-2 capable of producing high molecular weight levan at high temperature. J. Biotechnol..

